# The Brewers' Exhibition

**Published:** 1911-10-28

**Authors:** 


### The Brewers' Exhibition.

### SOME USEFUL APPLIANCES FOR HOSPITALS.

Although it might not at first sight appear that a Brewers' Exhibition would be prolific in ideas and appliances suitable for hospitals and other kindred institutions, a visit to the Royal Agricultural Hall cannot fail to disclose the fact that there are many useful appliances on view which are well worth the attention of hospital secretaries, matrons, or stewards.

### Ice Machinery.

During the past summer, and especially during the strike period, numerous hospital authorities would have welcomed the "W.K." ice machine, which is one of the most convenient and efficient ice-making machines on the market. This machine is already in great demand in hotels and restaurants, and possesses advantages which make it a most suitable appliance for hospitals. It is compact and easily worked; in three minutes ice can be obtained, and the construction is as simple as it could well be. The principle is that of creating a vacuum in conjunction with the presence of a powerful absorber, in this case the cheap and easily obtainable common commercial sulphuric acid. It is hardly necessary to point out what a great boon such a machine would be to a hospital in a country district, where ice is unobtainable, and there are many such places. Messrs. Hannefords, 124 The Minories, are the makers, and will quote special terms to hospitals.

### Imitation Marble.

The exhibit of the "Marosa" slabs of imitation marble is not only pleasing to the eye, but of direct utilitarian interest. These beautifully polished slabs are now being used in hospitals, theatres, bath-rooms, etc., and have advantages both æsthetic and aseptic over tiling, while they are, of course, much cheaper than marble. The "Marosa" slab is easily fixed to plaster, wood, or brick, and it may be cut or drilled without fear of splitting the material; a backing of fibre network prevents an unsightly hole appearing should a smash occur. "Marosa" makes a wall covering that is easily kept clean, is very durable, and low in cost. The manufacturers are Messrs. D. Anderson & Son, Ltd., Roach Road Works, Old Ford, E.

### Cleansing Apparatus.

A product which combines all the essential properties of soap, soda, scrubbing brush, and disinfectant seems worth the notice of hospital matrons. The makers claim that "Tarsap" is such a preparation, and a practical trial goes far to prove the justice of their claims. A quarter of a pint of this disinfectant cleanser added to a 3-gallon pail of water, either hot or cold, is more effective and less laborious than scrubbing with soap, and also less costly. At the same stand can be seen the "Alpha" spraying machine, which is one of the simplest sprayers yet introduced. Simple in construction and of non-corrodible material, the "Alpha" is ideal for disinfecting wards and sick rooms. By means of an ordinary bicycle pump these sprayers are readily charged, so that the whole of their contents may be distributed with ease. A convenient size can be purchased for 17s. 6d.

Hygienic syphons are now attaining an important vogue, and those made by P. W. Taylor & Co., of the Hop Exchange, S.E., are not only hygienic, but of simple construction. The parts are readily detached, and the porcelain spout, which replaces the simple cap they are sent out with, is retained by the user. This firm also makes patent tap jars, which will be found of great use in dispensaries and stores.

The Berkefeld filter is now so well known and so generally employed that we need only draw attention to them to mention that the firm has several modifications suitable for households, whether sufficient pressure is available or not, and also a travellers' pump filter. As to their efficiency, we are justified in concluding that these Berkefeld filters afford a complete protection against the communication of waterborne diseases.

Most institutions find employment for fluids contained in casks, and it is not seldom a matter of difficulty to empty a cask without rendering the contents turbid by the disturbance of a sediment. Here the patent automatic cask-tilter, invented by Mr. W. Thompson, of 45 St. Paul's Road, N., will find its application. The tilter requires no fixing to the wall and acts automatically, ensuring the liquor being drawn clear to the last drop.

### Beverages.

Among the numerous exhibits of all sorts of beverages on view at the Agricultural Hall we may claim attention for the Devon Cidro, one of the few palatable non-alcoholic drinks now on the market. It is made by the Teign Cider Co., of Netherton, Devon, and possesses an agreeable sparkling flavour; it is retailed at about 2d. a bottle. A word may be said of the Turtle Soup Tablets made by John Lusty & Co., Parnham Street, Limehouse, E. These tablets are quickly made into an appetising and stimulating soup, which is not without considerable nutritive properties, and has undoubtedly a superior flavour to many of the essences and extracts widely sold. The tablets cost 3d. each.

The makers of the long-established Idris mineral-waters are showing a new tonic wine, known as Karn wine, which is prepared from the soundest materials and contains extracts of meat and of malt.

A visit to this, the thirty-third annual trade gathering of brewers, distillers, mineral-water manufacturers, and allied trades is not without interest to readers of The Hospital, as we have shown, while the numerous competitions for malting barley, hops, beer, cider, etc., have a value of their own in stimulating farmers and producers.

